# Comprehensive characterization of copy number variation (CNV) called from array, long- and short-read data

**DOI:** 10.1186/s12864-021-08082-3

**Published:** 2021-11-17

**Authors:** Ksenia Lavrichenko, Stefan Johansson, Inge Jonassen

**Affiliations:** 1 Computational Biology Unit, University of Bergen, Bergen, Norway; 2 Department of Clinical Science, University of Bergen, Bergen, Norway; 3 Department of Medical Genetics, Haukeland University Hospital, Bergen, Norway

**Keywords:** CNV, Microarrays, Short reads, Long reads, Genome in a Bottle

## Abstract

**Background:**

SNP arrays, short- and long-read genome sequencing are genome-wide high-throughput technologies that may be used to assay copy number variants (CNVs) in a personal genome. Each of these technologies comes with its own limitations and biases, many of which are well-known, but not all of them are thoroughly quantified.

**Results:**

We assembled an ensemble of public datasets of published CNV calls and raw data for the well-studied Genome in a Bottle individual NA12878. This assembly represents a variety of methods and pipelines used for CNV calling from array, short- and long-read technologies. We then performed cross-technology comparisons regarding their ability to call CNVs. Different from other studies, we refrained from using the golden standard. Instead, we attempted to validate the CNV calls by the raw data of each technology.

**Conclusions:**

Our study confirms that long-read platforms enable recalling CNVs in genomic regions inaccessible to arrays or short reads. We also found that the reproducibility of a CNV by different pipelines within each technology is strongly linked to other CNV evidence measures. Importantly, the three technologies show distinct public database frequency profiles, which differ depending on what technology the database was built on.

**Supplementary Information:**

The online version contains supplementary material available at (10.1186/s12864-021-08082-3).

## Background

Copy number variants (CNVs) are a subtype of structural variants in a genome, that are characterized by a change in the amount of genomic material through either a loss or a gain of DNA in case of a deletion or a duplication, respectively. CNVs are often defined as spanning at least 50 basepairs and have been shown to play important roles in disease and complex traits [1, 2].

SNP arrays have been used for large-scale cohort-wide CNV ascertainment [3, 4] since their introduction in the early 2000s [5–7]. The main principle behind CNV detection from SNP data is the use of probe intensity values, normalized against a reference, as a proxy for the total allele copy number. It is well recognized that the effect of an array platform as well as CNV caller and its parameter choice is substantial and results in low concordance between platforms and callers. High false positive rates and varying sensitivity are well-documented challenges for array-based CNV calling. [4, 8–10].

Short-read sequencing technology has seen a rapid development in the last decade [11–13]. Tools that leverage short-read data for calling CNVs use four methods: read depth, discordant read pairs, split reads and assembly [13] as well as various hybrids of these methods [14–16]. Compared to arrays, short reads enable a better digital estimation of copy numbers and improve the resolution for small variants (<1 kilobase). Moreover, they are not limited or biased by a probe design. Despite these advantages, short reads present a challenge for CNV calling due to their length, which, together with the complexity of the variant content, lead to wildly varying performance of callers [17–21].

Long-read genome sequencing offers both single basepair resolution and multiple kilobase-long reads, that span many variants in full length [22], is amplification-free, and allows to reduce sequence coverage bias [23]. The algorithms designed to call CNVs from long reads utilize both intra- and inter-read signatures, as well as *de novo* genome assembly [24]. The known weaknesses of long-read technologies include elevated error rates (up to 20%) as well as challenges with CNVs that exceed the length of the reads in size [25]. Lastly, there is room for improvement for the algorithms and formats which are in use in the maturing long-read field [26, 27].

In this study we ask how CNV calling compares between the three technologies - arrays, short reads and long reads. Given that the existing benchmarking sets are still incomplete [28], we aim at performing the technology comparisons independent of a reference.

Previous efforts towards the characterization of CNV sets with different technologies can be roughly divided into direct comparisons of two or more data types and multi-source integration, in which various data types are combined and contrasted to varying extent. Studies of the first kind typically perform pairwise comparisons of two technologies, relying on different CNV validation strategies, ranging from the use of a published reference [29], to genotypes from population cohorts [30], and assays in short- or long-read data [31]. To our knowledge, a systematic comparison of all three technologies, that does not rely on any (potentially incomplete) reference and includes cross-technology validation, is lacking. Multi-source integration studies compare multiple technologies to a multi-source compiled reference, using various orthogonal evidence for validation, such as: matches to known events in the Database of Genomic Variants (DGV) [32]; support from hybrid assembly results [33]; additional sequencing and genotyping in short-reads [34] or raw PacBio reads, short-read depth coverage and other orthogonal techniques [28]. These studies are on the bleeding edge of the field and are too resource-demanding for most projects, which makes it hard to relate their findings to individual single-technology datasets.

We compared array as well as short- and long-read technologies in their ability to discover CNVs in the human genome. To this end, we assembled a comprehensive set of datasets for the Genome in a Bottle individual of Northern European ancestry NA12878 [35] of both raw genomic data and published CNV calls for each of the three selected technologies. Our data collection was furthermore selected in such way that it could represent a wide range of platforms and CNV calling pipelines for each technology. This then enabled us to assess and quantify the previously identified biases for these technologies. We present a thorough unbiased comparison of genomic loci with CNV calls for each of the three technologies and highlight their most important features. As such, this will help to interpret existing CNV databases in a better context as well as inform future studies involving large-scale CNV assays.

## Results

### Characterization of CNV calls in NA12878 with arrays, long and short reads using a read-depth based score

**Fig. 1:**
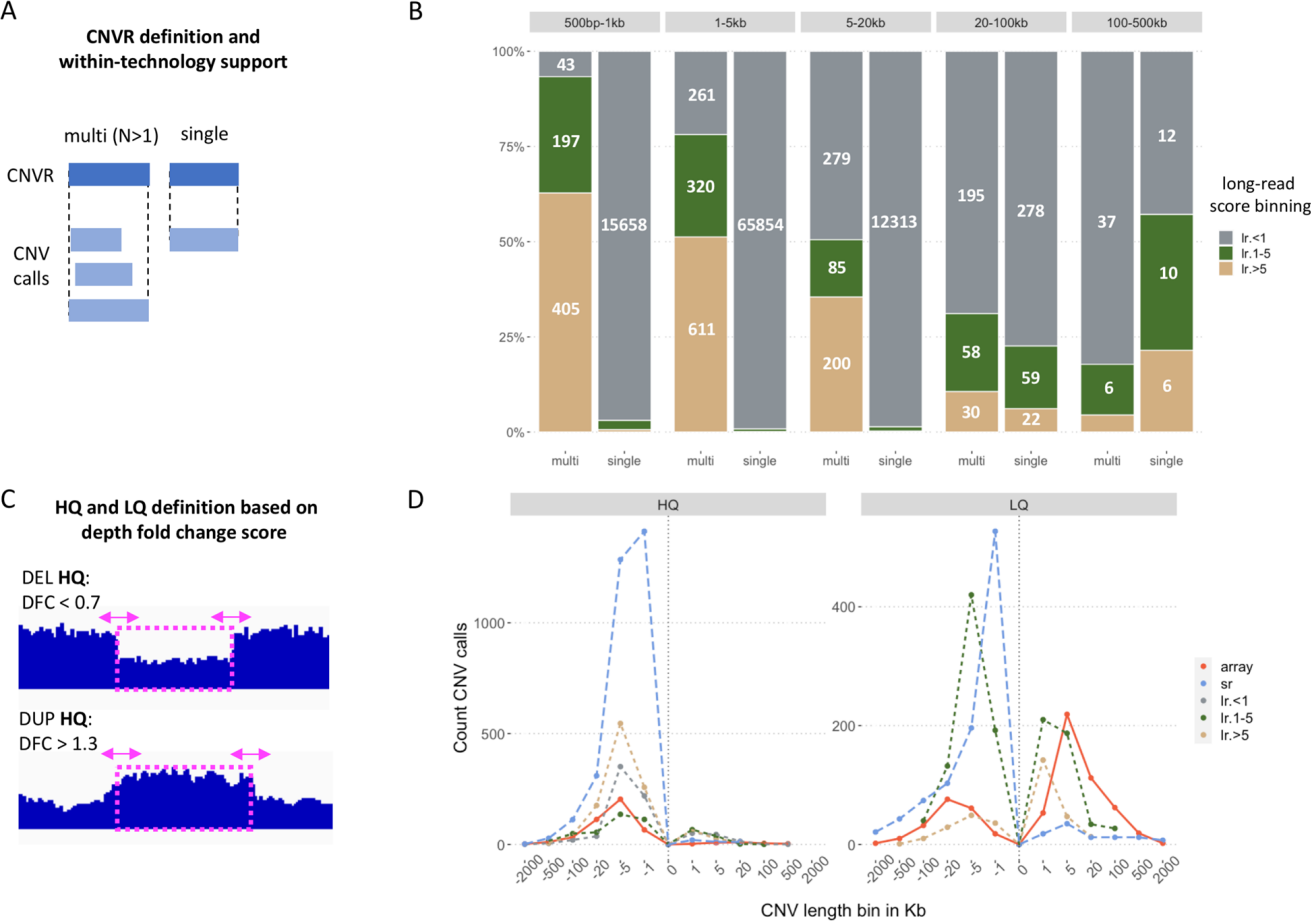
Defining and describing the CNVRs in terms of within-technology support, read depth fold change evidence, size and long-read intrinsic score distribution. **A**. CNVRs are defined by outermost upstream and downstream breakpoints for a set of CNVs of the same type. The within-technology support is defined as “single” when a CNVR is derived from a single CNV call of one of the datasets and otherwise as “multi”; **B**. Distribution of long-read CNVRs according to their length and the long-reads score bins (in grey, green and beige) and within-technology support (x-axis); **C**. For each CNV call a read depth fold change (DFC) score cutoff is used to define high quality (HQ) deletion (top) and duplication (bottom) with respect to support for a given CNV in a chosen short-read alignment. CNV calls that do not meet the defined DFC score thresholds are defined as low quality (LQ); **D**. Density plot (counts) for deletions (indicated with negative values on the x-axis) and duplications (positive values on the x-axis), across CNV call sizes (x-axis ticks), DFC score-based quality bins (left and right panel) with arrays shown in red, short reads in blue and long reads in grey, green and beige (for the long-read score binning as in B). The “long-read score <1” (lr. <1 in gray) category is omitted in the right panel for scaling purposes. Technologies: array - array, lr - long reads, sr - short reads

In this study we investigated and contrasted specific characteristics of CNV calls, derived from three different technologies - SNP arrays, short-read and long-read sequencing. To this end we assembled a large set of CNV calls for the well-studied NIST Genome in A Bottle individual of Northern European ancestry NA12878 [35]. For each of the three technologies we compiled callsets including published CNV calls, consensus calls and calls we made in-house from the public data for this individual (Table 1 and Supplementary file 1). This allowed us to construct a balanced representation of a broad range of CNV calling methods and platforms. Of note, for long-read technology we were able to include both raw [36] and error-corrected reads [34]. We then further collapsed redundant calls for each technology into CNV regions (CNVRs) using the outermost breakpoints to define the new start and end coordinates (Fig. 1A and Supplementary file 2).

**Table 1:** Summary of the CNV datasets and non-redundant CNVRs for each technology (length >500bp)

Source	CNV calling tools	Method summary	Ref.	Del.(n)	Dup.(n)	Del./Dup.
**CNV regions, array**	merge	-	CNVR	666	533	1.25
Illumina arrays^1^	PennCNV, cnvPartition [37], Nexus [38]	I, AR	[4]	93 222	126 37	0.74 6
Affymetrix SNP 6.0	PennCNV [39], Birdsuite [40]	I, AR	[7]	73	36	2.03
CytoScan HD	apt-copynumber-cyto [41]	I, AR	our data	105	25	4.2
HapMap II genotypes	GADA [42], custom	I, genotyping	[43]	460	360	1.28
**CNV regions, long reads**	merge	-	CNVR	74156	23959	3.1
ECR/RR PacBio CNVs^2^	PBHoney [44]-v1.3.1, cm-v1.3.1/v1.3.2, assembly	intra-read discordance, soft-clipped/unmapped tails	[34]	1925	NA	NA
ECR PacBio RR PacBio Oxford Nanopore	SVIM [45]	inter-/intra-alignment signatures clustered classified	[34] [36] [46]	1235 4265 71179	799 22718 1436	1.54 0.19 49.56
**CNV regions, short reads**	merge	-	CNVR	12932	309	41.85
Published CNVs, >30x	svclassify [47]	SVM classifier	[47]	2502	NA	NA
Published CNVs, 50x	MetaSV [48]	ensemble of RP, SR, RD, JM, soft-clipped tails^3^	[48]	11748	NA	NA
1000genomes III, >7x	BreakDancer [49], CNVnator [50], Delly [14], VariationHunter [51], GenomeSTRiP [52]	RP, SR, RD, population-scale genotyping	[12]	921	6	153.5
	Delly	RP, SR		2611	122	21.4
Illumina Platinum, >30x	GRIDSS [16]	genome-wide assembly, RP, SR	[53]	2278	181	12.58
	Manta [15]	local assembly, RP, SR		2581	137	18.83

^1^Selected platforms: CytoSNP850, Omni25, Omni5, OmniExpress; I, intensity; AR, allelic ratio

^2^ECR, Error-corrected reads; RR, Raw reads; v, blasr version; cm, custom method

^3^RP, read pairs; SR, split reads; RD, read depth; JM, junction mapping

Bold indicates the aggregated CNV regions based on all listed datasets for each respective technology

As short reads have been extensively examined with respect to their capacity to call CNVs (and other types of structural variants) [54], we focused our investigations on array and long-read technologies, and included short reads into comparisons in order to make it possible to relate our results to those of existing benchmarks. However, due to the known high false positive rates in CNV calls from short reads [13], we only included those calls that passed the default quality criteria of each respective method in the short-read CNV datasets. In contrast, for arrays and long reads, we retained all calls in the data, binning and filtering them by various quality metrics in the analyses performed in this study.

Since each method and callset reports different types of score metrics, and some lack a score altogether, we sought to annotate all CNV calls with a common scoring scheme. To this end we used duphold [55], a tool that, given a short-read alignment, provides a *read depth fold change* (DFC) score between each CNV locus and its immediate flanking regions. For CNVRs, the DFC is the median value of the constituting CNV calls. We then annotated each CNV call (and CNVR) as either High Quality (HQ) or Low Quality (LQ) using the DFC score thresholds recommended by the duphold tool developer (Fig. 1C). We refer to the SVIM caller [45] quality score as long-read *intrinsic quality score* and use it for binning long-read CNVRs (CNVRs with score <1, CNVRs with 1 ≤ score<5 and CNVRs with score ≥ 5).

As was expected, most pipelines tended to call more deletions than duplications (Table 1), with the largest difference between deletions and duplications being for short reads [56] and a more balanced representation in arrays (mostly due to the balanced set of CNV genotypes from the HapMap Consortium [43]). Interestingly, for long reads, the callsets based on raw reads showed varying and highly unbalanced ratios of deletions to duplications, while callset from the error-corrected PacBio reads had this ratio closer to one.

Furthermore, we found that for long-read CNVs, the *intrinsic quality score* agreed well, both with within-technology support (Fig. 1B.) and the DFC score (HQ, LQ, Suppl. Fig. S1). The SVIM caller reported much less CNVs (and more CNVs with higher scores) for the error-corrected PacBio reads [57] than for both raw PacBio [36] and Nanopore reads [46]. The two raw long-read datasets produced high numbers of CNV calls with a very low score (<1), which mainly fell into the size of <20Kb and were only supported by a single dataset. The effect of long-read error correction has been studied primarily with regards to its effect on downstream *de novo* genome assembly [58]. Pendleton and colleagues [34] reported counts for overlaps of deletions and mobile element insertions between raw and error-corrected PacBio reads (Suppl. Fig. 6 in [34]), but a thorough investigation of the impact of error correction on calling of structural variants is still missing. Several studies have assessed the effect of error correction on alignment rates, finding results to depend on the error-correcting method used [59]. This would in turn likely carry over to the accuracy of downstream CNV calling.

To further study the impact of array design on a CNV calls profile, we compared the density of array probes within the CNVRs of each technology. The array-derived CNVRs, on average, spanned more probes in all chip designs considered (Suppl. Fig. S2A), compared to those from the two sequencing technologies. In all three technologies, the CNVRs with higher within-technology support systematically spanned more probes along the genome than those CNVRs, supported by only one dataset (singletons). If CNVRs with more within-technology support are systematically longer than singleton CNVRs, that would explain why they also span more probes. This was the case for array-derived CNVRs (median length of 9.8Kb of the multi-supported vs. 3Kb of singleton CNVR, *P*-value < 2.2e-16, Wilcoxon-rank test), while the opposite held true for both long reads (2.54Kb vs. 3.35Kb, *P*-value=0.04) and short reads (0.34Kb vs. 0.41Kb, *P*-value=9.3e-14). This means that, while being on average shorter in basepair length, CNVRs based on the two sequencing technologies with better within-technology support, spanned regions with higher array probe density than singleton CNVRs did.

Next, we studied the percentage of CNVRs that had zero array probe coverage in a selected subset of array chips (Suppl. Fig. S2B). For array CNVRs, this percentage was notably larger for singleton CNVRs. In other words, CNVRs supported by only one chip design (singletons) typically covered regions where the other chip designs had no probes. On the contrary, CNVRs with support from multiple data sources based on long-reads more often were completely void of array probes in comparison to singleton CNVRs. This demonstrates that the long-read based CNVRs - and especially those with support from multiple callsets - often are in regions avoided when designing chips. Short-read CNVRs had nearly identical profiles in all chip designs (except Omni5) and showed the highest percentage of calls not covered by any probes, consistent with the larger number of shorter calls in the short-read datasets (Fig. 1D).

### Impact of quality scores on the definition and composition of CNV loci based on calls from all technologies

**Fig. 2:**
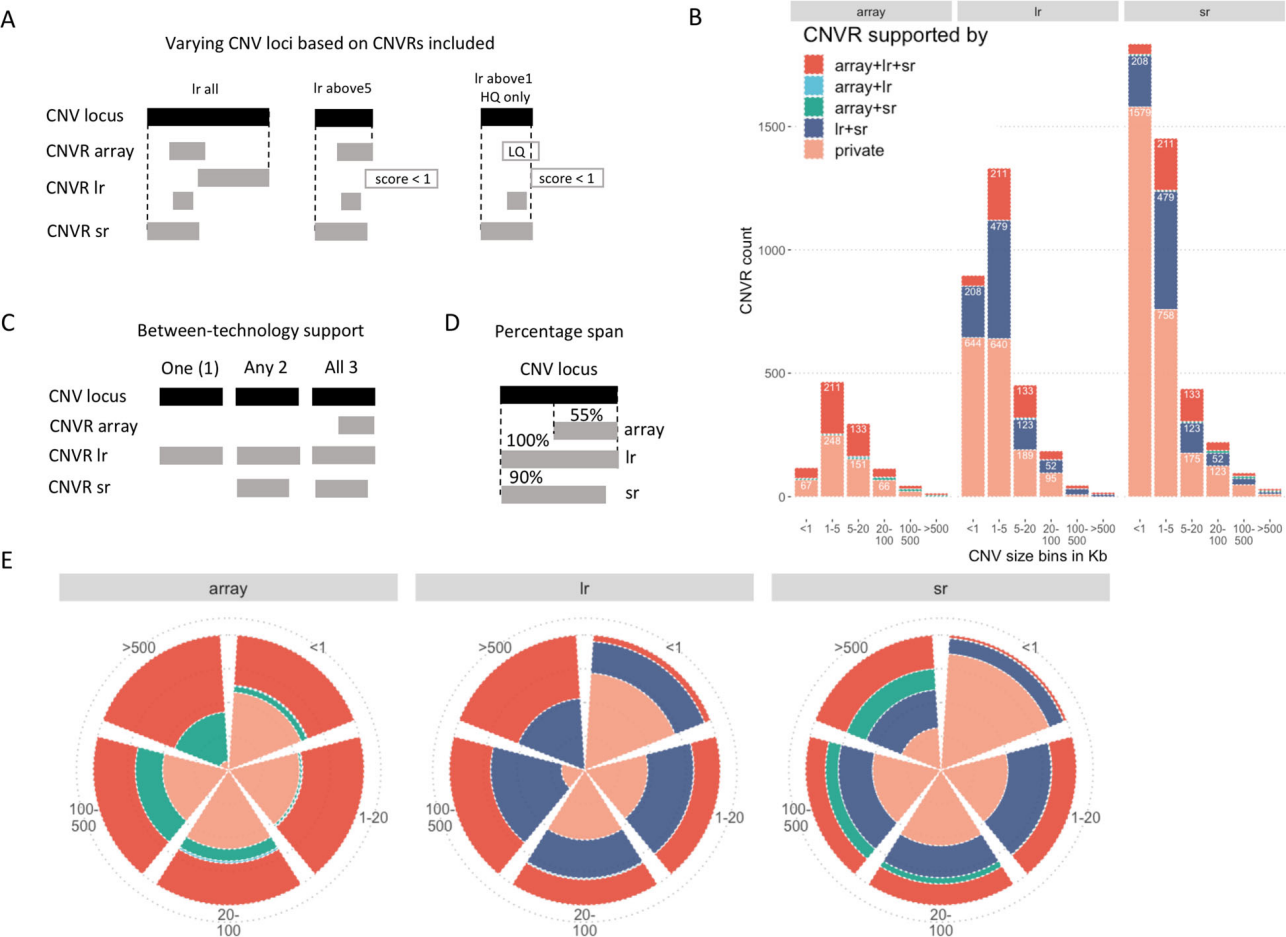
Definition of CNV loci, their composition and percentage span by CNVRs. **A**. CNV loci are defined by outermost breakpoints for a set of CNVRs of the same type. Depending on the CNVRs included in each technology, the resulting CNV loci boundaries will vary; left to right: all technologies CNVRs included, long-read CNVR filtered by intrinsic score >5 (boundary has changed because one long-read CNVR is no longer included due to its low score, indicated by hollow rectangle), long-read CNVR filtered by intrinsic score >1 and only HQ CNVRs included for all three technologies (boundary has changed again since now also an LQ array CNVR is not included, hollow top rectangle); **B**. Histogram of CNVR counts (using CNV loci for long-read score > 1 set as representative) binned by size (x-axis) and a list of supporting technologies for array, long-read and short-read, respectively; **C**. The between-technology support is defined as the number of technologies having a CNVR in the given CNVL (one, two or three); **D**. To compare sizes of constituting CNVRs for each CNV locus, the percentage span of CNV locus is calculated for each technology CNVR, e.g., length CNVR/length CNV locus × 100; **E**. Same CNVRs as in panel B, but visualized as proportions rather than counts. Color legend shared with panel B

To assess the concordance of CNVRs detected between technologies we constructed a non-redundant set of genomic loci across all three technologies, hereafter labelled CNV loci, representing the union of CNVRs (from all technologies) at a given locus (Fig. 2A). To facilitate comparisons of the quality of constituting CNVRs on these CNV loci, we created three gradually more stringent long-read CNVR sets filtered by *intrinsic quality scores*, which resulted in slightly different CNV loci (Fig. 2A). We complemented it by creating CNV loci for only High Quality (HQ) CNVRs for each technology and *intrinsic quality score* >1 (above1_HQ), representing a more stringent filtering strategy (Fig. 2A and Suppl. Fig. S3). We then used the resulting CNV loci to assess the between-technology support for each locus, either counting or explicitly listing the technologies with CNVRs present within the locus (Fig. 2B, C, E and Suppl. Fig. S3).

The percentage of CNV loci private to long reads varies with the choice of long-read *intrinsic quality score* cutoff, due to abundant low-score calls (Fig. 1B). Short reads show the highest percentage of private CNVRs. On the contrary, the array calls tend to agree with at least one other technology, but even more often with CNVRs from both long and short reads. Comparing the various quality CNV loci (Suppl. Fig. S3), it is notable that the proportion of CNV loci exclusively shared between array and long-read CNVRs is high when considering an unfiltered long-read dataset (Suppl. Fig. S3, left-most pie chart on top). Once a long-read score filtering is applied, it drops to a low percentage. At least a fraction of these could be common low-quality CNV calls, shared between long-read and array CNVRs. Once removed from the long reads, they shift to the “private” category for arrays (Suppl. Fig. S3, array “above1” panel). Further indirectly supporting this notion is the fact that the majority of “private” array calls disappear when further setting a requirement for only HQ calls from each technology (Suppl. Fig. S3, array “above1_HQ” panel).

The percentage of span of a CNV locus by CNVRs in each technology is another indirect quality indication (Fig. 2D and Suppl. Fig. S4). In a *bona fide* CNV locus, the breakpoints of CNVRs from different technologies would be relatively close and, consequently, CNVRs will be spanning most - or a high percentage - of their corresponding CNV locus (examples of *bona fide* deletion and duplication on Suppl. Fig. S5 and Suppl. Fig. S6, respectively). At the other end of the spectrum, more challenging genomic regions contain repeats that cause problematic mapping of reads and resulting erroneous calls. Such regions are known to be problematic for all technologies [8, 54] and have poor coverage in many array chips designs. Thus, the resulting CNVRs will vary much more in their breakpoints and number as well as in the distance between them. This can be observed for very large CNV loci (>500Kb) that tend to have a low percentage of overlap between the constituting CNVRs, meaning that these loci are likely driven by a single CNVR. We expect they could be artefacts in one of the sequencing technologies, because array-based calls are more robust for very large calls. We examined eight large CNV loci from the “long-read score >1” category that were found in all three technologies and had low percentage spans by constitutive CNVRs. In seven of the eight cases we found that they were driven by a large low quality CNVR from short reads, and were gone in the more stringent “above1_HQ” CNV loci set. The remaining CNV locus of these eight was driven by a high-quality array deletion CNVR and was also supported by the visible Log R Ratio distribution shift below zero in the array signal data (as expected for a *bona fide* deletion call). More generally, removing low quality (LQ) CNVs for all technologies, resulted in a much-reduced number of loci with a low percentage span (Suppl. Fig. S4C).

### Technology-specific support for CNVRs is consistent with other quality cues

**Fig. 3:**
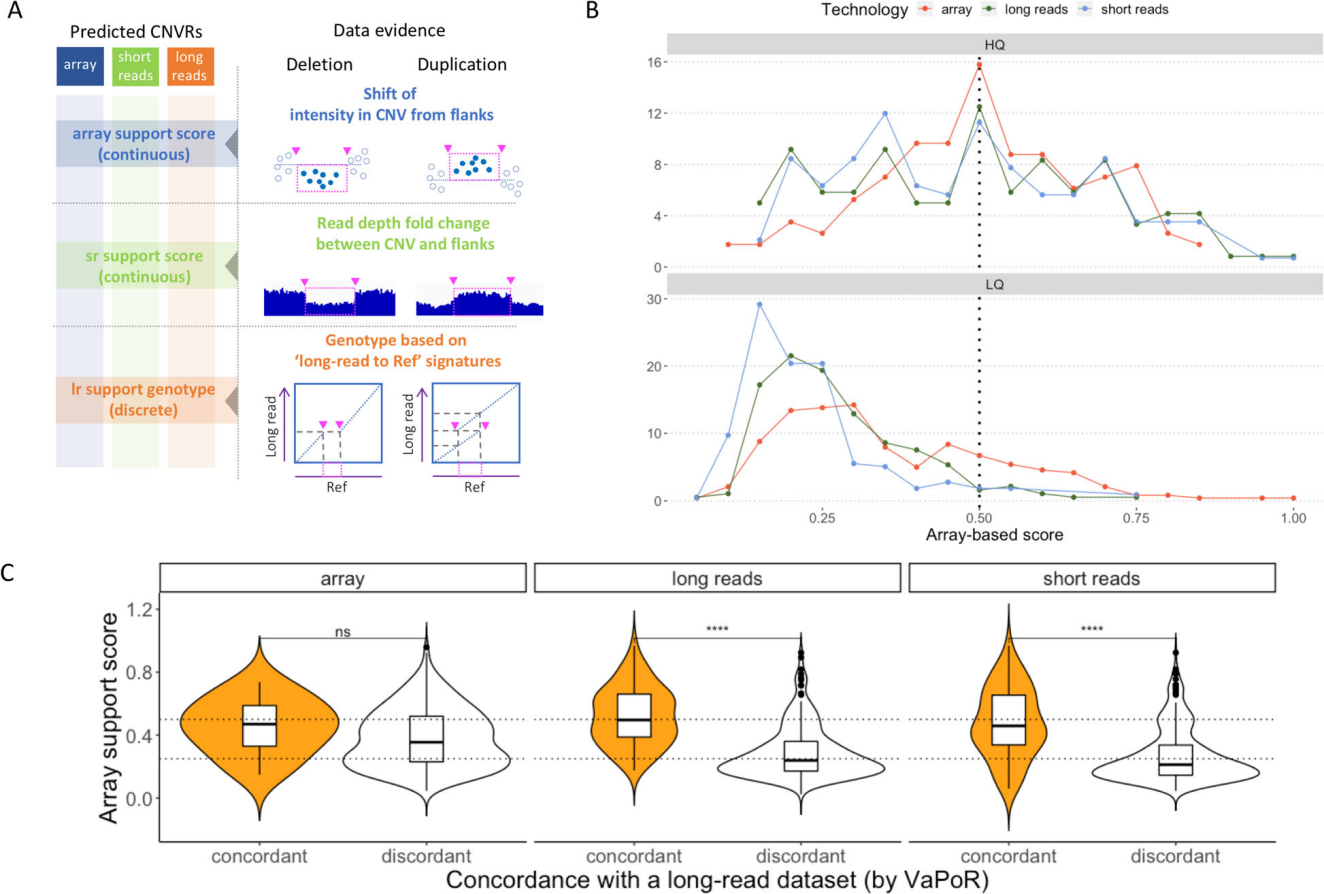
Cross-technology support relation to quality cues. **A**. Cross-technology data evidence collection. For each CNVR in arrays, long- and short read set, the raw data in all three technologies is assayed for evidence of support. Array signal in the probes within CNVR is compared to the flanking regions, resulting in a distance metric; Read depth fold change score is used as evidence for the short-read data; Long-read data is assayed as shown on the dot plots, for evidence of genotypes. **B**. Normalized density plot with the array-based support score on x-axis (the score represents the distance between LRR distribution of the probes in the CNV versus flanking regions, the larger the distance, the more support for a CNV), split by DFC score bins (High and Low Quality) and colored by technologies; **C**. The violin plots showing distribution of array-based support score for CNVRs grouped by support derived from assaying the long-read data, with “concordant” group denoting CNVRs, for which the long reads indicated concordance with the presence of the variant, while for the “discordant” group the was no such support from the long-read data

In the first sections of this paper we used quality labels, High Quality (HQ) and Low Quality (LQ), defined by a short-read depth fold change (DFC) score (Fig. 1C). We extended on this by systematically investigating the support in each technology raw data for every single CNVR, regardless of its origin (Fig. 3A).

For each CNVR, we sought to see if it is supported across technologies (arrays, short reads, long reads). For this, we picked one dataset to represent each technology and assayed its raw data. For the given CNVR we analyzed if it is supported by the chosen raw data set (Fig. 3A). For array raw data, we calculated the intensity shift in the CNVR region relative to its flanks. For short-read data, we calculated the *depth fold change* (DFC). For long-read data, we used the VaPoR method [60] that aligns the long reads to a reference as well as to a reference modified in accordance with a predicted CNV (e.g., with a segment removed for a called deletion). We then used the call from the VaPoR tool to determine which has more support (the reference or the modified reference). For each CNVR we then got a number (for arrays and short reads) or a genotype call with the highest likelihood (for long reads) (Fig. 3A and Supplementary file 3), which we further interpreted as supporting evidence (genotypes 1/1 and 0/1) or the lack of supporting evidence from the long reads (genotype 0/0 or no assigned genotype). This allowed us to study if a CNVR well-supported by short reads is also well-supported by long reads etc. The most useful way we found to perform this comparison was to divide the CNVRs into high-quality and low-quality groups based on one technology and to study the distribution of continuous scores.

**Short-read and array data evidence** In Fig. 3 panel B we summarized our findings for CNVRs from all three technologies (indicated by different color lines). They are grouped into low and high quality based on their support in short-read data. CNVRs with little support (LQ) in short-read data were found to also have little support in array data as the distribution of array-based scores is shifted to the left (low scores) in the low-quality panel. The same holds true for CNVRs called by all three technologies (lines with the different colors).

**Long-read and array data evidence** In Fig. 3 panel C all CNVRs are considered for each technology, and grouped by support in the chosen long-read dataset, based on long reads support for the reference (“discordant”) or the modified reference (“concordant”). We then for each CNVR retrieved how well it is supported by the chosen array dataset (Supplementary file 4). We find that the CNVRs based on short-read or long-read technologies show agreement between array and long-read support. In other words, CNVRs labelled as “concordant” with the presence of the CNV from the chosen long-read dataset also have higher support in the array data, and the other way around. However, for CNVRs called by arrays (leftmost panel), there is no such pattern. Indeed, the array-based CNVRs tend to have higher support in the array dataset regardless of the status in the long-read evidence, pointing towards array-specific properties of these calls.

**Short-read and long-read data evidence** Finally, we looked at the distribution of the short-read based DFC score within the support label groups (concordant/discordant) derived from long reads for each CNVR (Suppl. Fig. S7A). There were no duplications in arrays that were supported by long-read data evidence. The deletions that were supported by the long-read data in most cases had DFC scores below 1 (support for a reduced read depth in short-read data within the given CNVR). Consistently with that, deletions, as well as duplications, that were not supported by long-read data had a clear peak in DFC score around 1 (no read depth fold change in either direction in short-read data). These observations held true for CNVRs originating from all three technologies.

Interestingly, there was a substantial fraction of deletions in the long-read “discordant” group that was supported by the short-read data (DFC score shifted below 1 - reduced read depth), most prominently for array deletions. We conclude that the CNVRs (regardless of their technology of origin) that are supported by long-read data are also likely to be strongly supported by short-read data. The opposite trend only held true for duplications, but not for deletions.

**Short-read data evidence and other quality metrics** To follow up, we investigated how DFC score relates to other quality metrics. In order to do so, we first studied how this score is distributed, specifically in the long-read CNVRs, binned on basis of their intrinsic quality score (Suppl. Fig. S7B). We observed a very consistent trend in which the vast majority of low-scored long-read CNVRs lacked support in short-reads (no read depth fold change detected). The more stringent the score filtering was, the larger the fraction of CNVRs that showed short-read data support. This effect was observed for both deletions and duplications, but more pronounced for deletions. Next, we investigated the between-technology concordance grouping and observed that CNV loci identified by a single technology tended to be less supported by short-read data (Suppl. Fig. S7C). Again, the more technologies supported a CNV locus, the more likely it was to observe stronger short-read support. This was very consistent across technologies and valid for both deletions and duplications, with the exception of private short-read based deletions. These tended to have a higher support based on DFC score in the selected representative short-read dataset.

For an overview of all the evidence we collected for each technology, we extracted a subset of CNVRs that possessed intrinsic scores, i.e., from datasets in which we called CNVs in-house from array (PennCNV score) and short-reads (GRIDSS score). We found that the indirect quality categorization obtained through grouping by within-technology support (single/multi) was in good concordance with all considered metrics (Suppl. Fig. S8). This means that CNVRs that were detected in multiple datasets within each technology tended to have higher scores than singleton CNVRs across all metrics and all technologies.

### Public database frequencies of CNVs

We aimed to explore whether we could observe any biases in public CNV database frequencies, using our multi-technology CNVR collection. We wondered if a CNVR that is supported by several datasets within a technology, would, for example, be more likely to have a higher frequency in a database, than a CNVR that is only supported by a single dataset (singleton)? Furthermore, could this behavior change, if a different public database is used to retrieve the CNV frequencies?

We therefore selected commonly used and relatively large databases, ranging from a database that contains a large fraction of CNVs derived from array-based studies (the Database of Genomic Variants (DGV) [32], to purely short-read based databases (gnomAD (GD) [61] and Ira M. Hall lab database (IMH) [62]), as well as the Deciphering Developmental Disorders (DDD) [63] controls database, containing both short-read based (deep whole-exome sequencing) and array-based (aCGH) calls. For every CNVR, we checked its overlap (at least at 50% of its length) with CNVs in each database. In case overlap was observed, we retrieved the frequency of the respective CNV in that database.

**Fig. 4:**
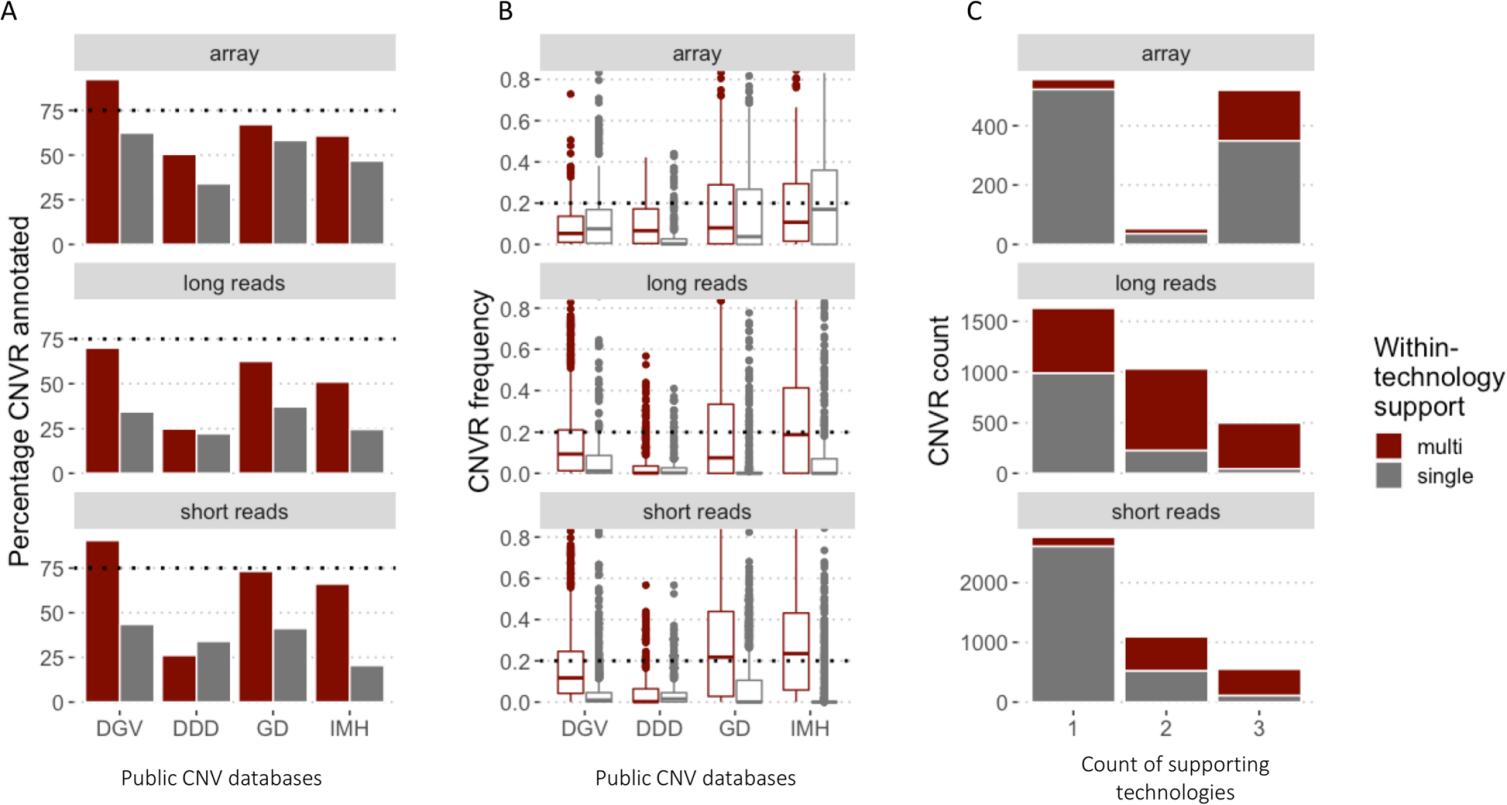
CNV presence and frequencies in public databases. Within and between technology support. **A**. Percentage of CNVRs present (at 50% overlap) in public databases; **B**. Frequencies of CNVRs in public databases (at 50% overlap). DGV - Database of Genomic Variants, DDD - Deciphering Developmental Disorders database, GD - gnomAD and IMH - Ira M. Hall lab database; **C**. Relation of between-technology (x-axis) and within-technology (color fill) support

We found that CNVRs with support in multiple datasets tended to be present in public databases more often than singletons (Fig. 4A). This was observed for almost all technologies in all databases, except in the case of short-read based CNVRs. Here the singleton CNVRs were more often present in the DDD database than the multi-supported ones. Among the four databases considered, the DDD database had the lowest percentage of CNVRs matching all three technologies, while the DGV database had the highest percentage.

The database frequency profiles were most similar for the two sequencing technologies (Fig. 4B). The largest differences between singleton and multi-supported CNVR frequencies were observed for the short-read based databases - GD and IMH. The array-based CNVRs exhibited a very different (and sometimes reverted) pattern of database frequencies as well as their relation to within-technology support.

Overall, long-read CNVRs were detected less often in the public databases than CNVRs coming from the other two technologies and those that were detected had lower frequencies compared to the short-read CNVRs.

In terms of between-technology concordance, CNV loci that were detected by all three technologies had the highest percentage of multi-supported CNVRs (Fig. 4C) - that is, these CNVRs are also supported by multiple datasets within each technology. On the other hand, private CNV loci were dominated by singleton CNVRs for array and short reads, but not for long reads. CNVRs based on long reads had a substantially higher percentage of CNV loci with multiple within-technology support across all categories of between-technology support.

## Discussion

To the best of our knowledge, this is the first study that explicitly cross-assays CNV calls based on array, short- and long-read data, investigating possible signals in the raw data for each of the technologies. By including a variety of published and in-house generated CNV calls our analysis encompasses a wide representation of popular CNV calling platforms and analysis pipelines for each technology. This allowed us to focus on technological rather than methodological differences between datasets. We defined a number of simple but informative metrics, such as within- and between-technology support, long-read score and short-read depth fold change score bins, which we then used to quantify and compare the evidence found in the raw data from each technology.

Having studied the within- and between-technology support for CNVs, we wanted to further understand what genomic features might underlie the difference between the support from the respective data for each technology.

One of the most relevant genomic features for CNV calling is the presence of repeats at the breakpoints, especially segmental duplications [64], which influence the mappability (Suppl. Fig. S9, top panel). We found that among the three technologies, short-read CNVs showed the largest differences in coverage by segmental duplications at the breakpoints among the within-technology support categories (Suppl Fig. S8, lower panel), e.g., more supported CNVs tended to have lower segmental duplication coverage than less supported ones. This tendency was much less pronounced but still visible for the long-read CNVs and not at all for arrays.

On the other hand, CNVs with higher within-technology support tended to have flanking repeats around breakpoints for both short- and long-read sequencing technologies more often than less supported CNVs (Suppl. Fig S9, top panel).

Finally, we noted that in loci with no flanking repeats, long-read derived CNVs had significantly higher GC-content around the breakpoints than short-read ones (mean 0.56 vs. 0.51, Wilcoxon-rank test, *P*-value=9.5e-06, Suppl. Fig. S10). This supports the notion that long reads can be more efficiently anchored due to the possibility of spanning more unique subsequences in the genome, than short reads or array probes.

We observed that array calls often tended to agree with one or both sequencing technologies. Li and colleagues [31] performed deep analysis of array- and NGS-based CNV calling in 254 individuals, limited to losses in genic regions. They found less than 30% overlap between the two deletion sets they studied, while almost all array-based deletions had lower read depth in the NGS data. Moreover, 88% of those deletions had sequence support at the breakpoints, when checking the direct support from the reads, similar to our own observations. We expanded this idea by performing a reciprocal assessment of array data support for the sequencing-based calls, both for deletions and duplications.

Zhou and colleagues [29] compared different strategies at low-coverage short-read WGS in the CNV detection capabilities of 17 published array platforms, using the 1000 genomes project CNV set as a golden standard. They found that short-read based WGS strategies detected drastically more reference CNVs and exhibited smaller percentages of CNVs that were not validated by reference overlap. The choice of the sequencing-based reference in this study is likely to introduce a bias in favor of short reads, which should be kept in mind when interpreting the results. We tried to avoid this by not using any “golden standard” but rather defining the CNV loci originating from all three technologies, with varying inclusion criteria based on carefully selected quality indicators.

We also observed that long-read data allows us to call CNVs in genomic regions which, due to repeats and problems with mappability, are less accessible to arrays and short reads. This leads to a distinctive profile of CNV calls derived from long-read data. Similar conclusions were drawn by Couldrey and colleagues [30], when they compared a PacBio-based set of CNVs to short-read variants. They noted an inherent difference in the detection scope of the two technologies, with fewer calls and a shift in size distribution towards the calling of shorter regions in PacBio. Next to this, there was an overall low overlap between the CNVs from the two technologies. Our investigation of the public database frequency profiles further demonstrated that short-read CNV frequencies correlate with long-read CNV frequencies to a varying extent, depending on the database used.

To tackle a heterogeneous assembly of fifteen CNV datasets for the same individual, we adopted the simplest aggregation strategy, namely, collapsing all overlapping CNV segments to the outermost breakpoints, separately for deletions and duplications. While this strategy creates a tractable set of non-redundant CNV loci, it is also susceptible to creating large artefact CNV regions, driven in most cases by a single lower quality call. One way to control for this is to be able to categorize CNV calls into likely high and low quality, before collapsing redundant CNVs in each locus. To this end we used the read depth fold change score, calculated by comparing the read depth of the CNV locus to that in the flanking regions. We used this and other quality metrics to bin CNV sets by quality and thus creating several versions of aggregated CNV regions and loci, ranging from loci more likely to have artefacts to those less likely to have them due to stringent quality cutoffs.

The caveat of using the read depth score from short-read alignment is that the evidence itself is then subject to the biases of the short-read technology. The use of different technologies allows to pinpoint these biases, which then would be expected to be observed only for short-read CNVs - exactly as we have seen with short-read CNVs being more often supported by read depth fold change scores, regardless of other quality categories. The two other metrics - array-based score and long-read support - were collected across all technologies datasets to provide additional angle on the quality, independent of the DFC score.

Finally, while the within- and between-technology support metrics allowed us to slice the dataset in meaningful ways, they are also subject to biases. For each technology, repeatedly called CNV regions can be both true calls with clear evidence in the raw data as well as common artefacts (such as many CNV calls in the vicinity of centromeres and telomeres), which warrants caution in the results interpretation. Additional details, such as the span of a CNV locus by the segments that constitute it, presence of both deletion and duplication calls within the same locus, assembly support from short or long reads, orthogonal technology data or validation by pedigree data may help to further delineate the more likely *bona fide* calls from artefacts.

The study design developed here allowed us to overcome the limitations of an incomplete or lacking “golden standard”. Moreover, given a range of emerging technologies, our design can be further extended to include new types of evidence. At the same time, our results can be readily interpreted in the context of the existing benchmarking studies on the same individual, providing a useful link for relative comparison between technologies. Other practical aspects, such as cost-effectiveness, availability or required computational resources will of course need to be taken into consideration and might have different weight depending on the study.

## Conclusions

Our results confirm that long reads call CNVs in regions not easily accessible to short reads or arrays, while short reads have the highest proportion of small private CNV calls (500bp-1Kb). The reproducibility of a CNV by different pipelines within each technology is strongly linked to all other support metrics studied here. Importantly, all three technologies show distinct public database frequency profiles, which also differ depending on what technology the database was primarily built on. Our study provides an unbiased comparison of the three technologies and both a method and a data collection that can be further explored and expanded on.

## Methods

### Data collection

We assembled five datasets for arrays, four datasets for long reads and six datasets for short reads, respectively, either using CNV calls already published or performing the CNV calling from the (mostly public) raw data in-house. All datasets are provided in the Supplementary file 1.

### Genome annotations

The following annotations were obtained from UCSC Table Browser for human genome build hg19:

- Segmental duplications (Repeats/ Segmental Dups/ genomicSuperDups)
- Centromeres and telomeres (Mapping and Sequencing/Chromosome Band)
- Mappability (Mapping and Sequencing/ wgEncodeCrgMapabilityAlign100mer.bw)

ENCODE Blacklisted region hg19 was obtained from [65]. The segmental duplication track was merged to collapse complex nested segmental duplication loci into larger contiguous segmental duplication regions (merged segDups).

### Data preprocessing

All CNV calls, alignment files and genome annotation have been done in, obtained or lifted over to UCSC hg19 genome build. PennCNV [39] was run with defaults on the raw array intensity files for the chips in the multi-array set and Affymetrix SNP6.0 data. As the published calls on multiple array did not have a copynumber annotation, two methods to add this information were used:

- The CNVRs were matched against three other array sets with percentage overlap selected so that no CNVR is annotated with two types (e.g., deletion and duplication), which was at the 70% reciprocal overlap. For the CNVs that met this criterion, a type was added from cognate overlapping segment with known copynumber;
- all CNVRs were mapped back to each chip and attempted to validate with PennCNV validate script. If validated, the copynumber was assigned to the cognate CNVR, otherwise left unknown.
- Only the copynumber-annotated subsets of CNVRs were taken forward for further analysis.

CNV calls from raw and polished PacBio reads as well as Nonopore reads were obtained with SVIM [45] (ngmlr aligner option) and only the deletions and duplications (interspersed and tandem) >500 bp were taken forward for analysis. Sv-callers [66] was run on the short-read alignment with defaults and only calls that passed the default quality requirements and >500 bp were taken forward. GRIDSS [16] developer R script was used to interpret the breakpoints from GRIDSS. Finally, each dataset was filtered against telomeres and centromeres as well as ENCODE blacklisted regions.

### Quality binning across platforms

Since each technology and each caller has its own score and more than half of the published callsets have no scores, we sought to add a scoring scheme that would be uniform for all technologies. To this end we used duphold [55], which, given a short reads alignment, calculates a depth fold change for a variant locus versus flanking regions. We annotated all callsets with duphold fold change scores and used suggested values of DHFFC < 0.7 and DHBFC > 1.3 to define a High Quality (HQ) deletion and duplication respectively. This provided a uniform scoring across technologies and a labelling of each CNV as either HQ or LQ.

### Aggregating CNVs within each technology

All sets were first merged to collapse redundant segments for the replicate and different callers in array sets, multiple calls in short and long read sets. Importantly, the dataset CNV sets were first split into HQ and LQ sets and then merged within each set. This was done to avoid low quality segments driving artefact CNV regions (CNVRs). For arrays, however, first the merging was done within each set and then split to HQ and LQ sets. This is due to redundant calls in arrays from replicates of the same sample and redundant calls by different callers from [4]. For each technology, a master set then was created, for deletions and duplications separately, merging once more, and annotating in how many sets a call was present, which represents the within technology support (“multiple/single” label and a count of supporting callsets for each CNVR). For long reads, the intrinsic quality score (SVIM score) was propagated by calculating a median across all merged segments.

### All-way CNV comparison between the technologies

In order to determine the common CNV loci across the three master call sets, e.g., aggregated CNVR in three technologies in high quality and law quality bins (six sets in total), we repeated the merging procedure with at least a single basepair overlap, for deletions and duplications separately. This produced the CNV locus, which then was used to track the shared CNVRs. To determine the influence of the intrinsic score filtering in long reads, we create three sets: all calls, calls with SVIM score >1 (above1) and calls with SVIM score >5 (above5). In addition, we created a fourth set with SVIM score >1 and only HQ CNVRs (above1_HQ). The above-described procedure of the merging was then repeated for so-defined sets.

### Score bins percentage span

In order to study to which extend the CNVRs overlap between the three technologies, we used a CNV locus as a reference for each call within, and reported a percentage of CNV locus covered for each CNVR, e.g., length (CNVR)/length (CNV locus) × 100. When multiple CNVRs from a technology were present in one CNV locus, we used their total length for percentage coverage calculation. Since HQ and LQ regions may overlap within each technology, we performed these calculations separately for each quality bin.

### Array probe coverage

In order to evaluate the coverage by array probes for all CNVR sets, we used bedtools (v2.29.2) [67] intersect to determine all markers falling within a respective CNVR and then counted the number of markers, reporting zero for no intersection. For this we used array probe maps from several chip designs from [4]: Illumina’s OmniExpress, CytoSNP850, Omni25, Omni5 as well as Affymetrix SNP 6.0 and CytoScan HD

### Array-based CNV evidence

To investigate to which extend raw array data support each given CNVR, we used Affymetrix SNP 6.0 data for one replica and extracted Log R Ratio values (LRR) for all probes within a CNVR and 50 probes in flanking regions immediately up- and downstream (flanks) using extract_snp_single.py script from SeeCiTe package [68]. We then summarized this data by comparing the LRR distributions in CNV and flanks by calculating Hellinger distance (ranging from 0 to 1) between the two distributions. The larger Hellinger distance reflects a shift of LRR in CNV from flanks and thus supports a del or a dup in the locus. Additionally, we calculated the median of the LRR within a CNV locus, as the evidence of the shift from the signal values – expected around -0.5 for a heterozygous deletion and around 0.3 for a duplication.

### Long-read based CNV evidence

For validation of CNVRs with the long read data, we used an SV validator tailored for PacBio technology called VaPoR [60]. For a list of SV regions in bed-like format the tool assays the PacBio read alignment and reports a number of metrics, among which the genotype of the proposed SV (VaPoR_GT in standard notation of 0/0, 0/1, 1/1 or NA). We consider variant-supporting genotypes 0/1 and 1/1 as “concordant” with the presence of the CNV, otherwise we label it “discordant”.

### Intrinsic scores for long-read based calls

Three of the four datasets for the long reads were produced by a SV caller SVIM [45], which provides a quality score, ranging 0 to 100. The score incorporates various types of support for an SV, but in the latest releases of the tool, the developers note that the formula puts more emphasis on the number of the supporting reads above any other features. The published consensus dataset [34] is a superset of calls produced using three different variant detection methods, with varied parameters, resulting in a total of seven approaches, one of which includes assembly. While there is no score associated with the calls, the number of approaches supporting each variant (max N=7) is provided, which we used here as a score and loosely normalized it to fit the SVIM score.

### Intrinsic scores for array-based calls

Since the published array callsets came without a quality score, we sought to extract scores from the calls we generated in house, using PennCNV [39] for the multiple array platform data as well as for the subset of published calls from [4] that were successfully validated by PennCNV.

### Intrinsic scores for short read-based calls

We used GRIDSS scores, which were available for CNVRs, that included CNV calls by GRIDSS. For the comparison with the other intrinsic scores (Supp. Fig. S7) we normalised the score, dividing it by 100.

### Public databases

AnnotSV [69] was used on all CNVR sets to retrieve the public database matches and frequencies. The four databases then were selected for the analysis.

### Genomic context annotation

AnnotSV [69] was used on all CNVR sets to annotate repeats and GC content in the regions flanking the CNVRs. Additionally, the percentage covered by segDups was calculated using bedtools (v2.29.2) [67] on merged non-redundant segDup track from UCSC. The average mappability score was produced with a bigWigAverageOverBed tool over the wgEncodeCrgMapabilityAlign100mer.bw.

## Supplementary Information

**Additional file 1** Unfiltered CNV datasets for the three technologies. Original datasets, annotated with duphold score and SVIM score (for long reads). The long-read datasets were included at a size cutoff of 500 bp, while the short-read ones at a cutoff of 100 bp. In the analyses only CNVs > 500 bp are considered.

**Additional file 2** CNVR and associated CNV loci. CNVR and associated CNV loci coordinates and scores for the four quality bins, based on long-read score (all, score >1, score >5) and long-read and short-read quality binning: score >1 and HQ.

**Additional file 3** VaPoR output for CNVRs. VaPoR tool outputs for each technology CNVRs (using error-corrected PacBio dataset)

**Additional file 4** Array score for subset of CNVRs. Array score for CNVRs that was possible to map to Affymetrix SNP 6.0 chip with span of more than 5 probes.

**Additional file 5** Supplementary figures. Supplementary figures S1-S10.

## Data Availability

The analysis and main figures code is available at github.com/aksenia/treetech. The datasets supporting the conclusions of this article are included within the article (and its additional files).
