# Supplementary material for: Comprehensive characterization of copy number variation (CNV) called from array, long- and short-read data
Source: BMC Genomics. 2021 Nov 17;22:826. doi: 10.1186/s12864-021-08082-3 (PMC8596897; doi:10.1186/s12864-021-08082-3)
Supplement: Supplementary file 5 — Additional file 5 Supplementary figures. Supplementary figures S1-S10. [file 12864_2021_8082_MOESM5_ESM.docx]

Supplementary figures S1-S10


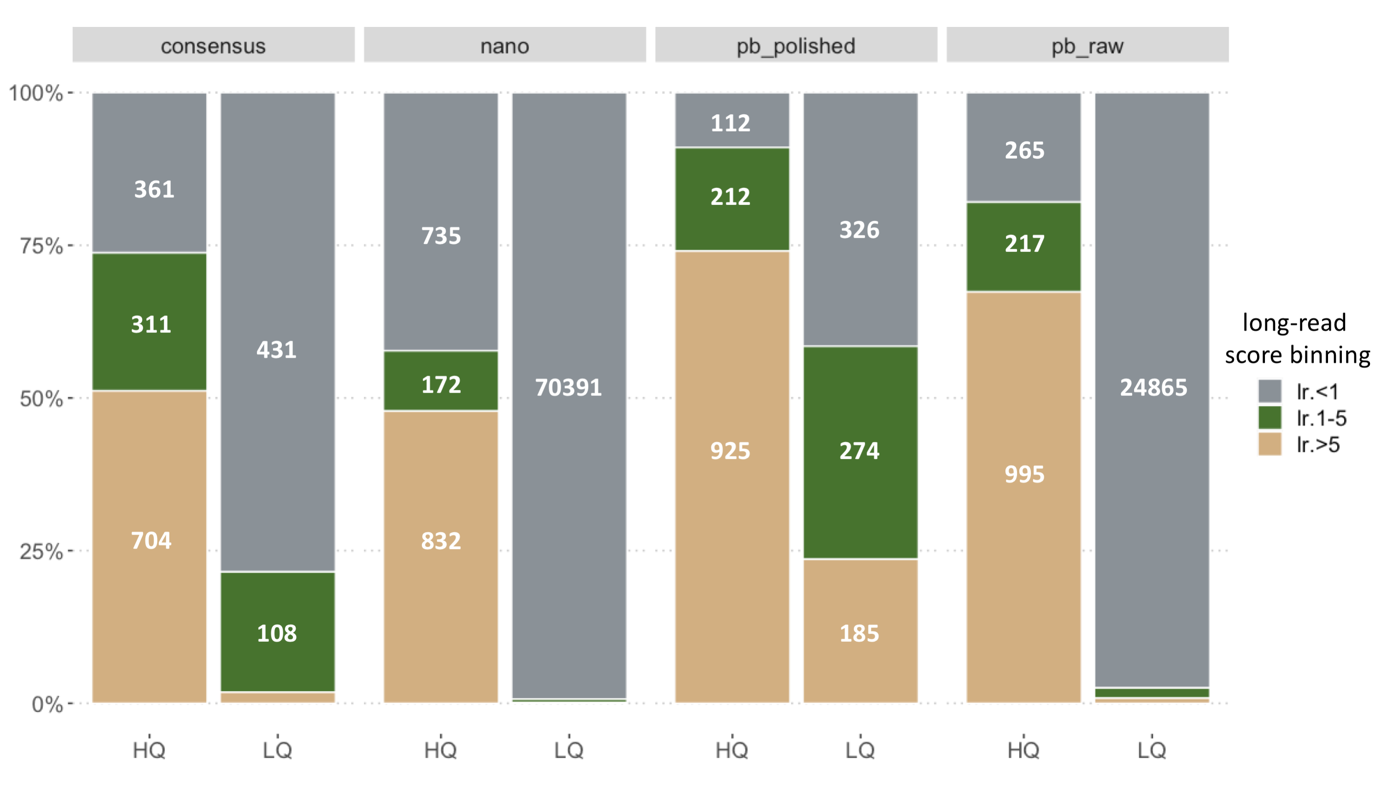


Figure S1. **Intrinsic score for long reads and DFC score quality binning.** Distribution of long-read CNVs per original dataset according to the long-reads score bins (in grey, green and beige) and DFC bins (x-axis). The low quality LQ bins consistently capture the CNVs that have very low intrinsic scores (<1, grey) in each of these datasets, while HQ bins are dominated by the higher scores (>5, beige). To note, the consensus dataset score is not provided by a caller. Instead, it is a simple scaled metric that reflects the number of methods (out of 7) that supported a CNV. This score behaves similarly with regards to the DFC bins consistency. DFC, depth fold change; HQ, high quality, LQ, law quality


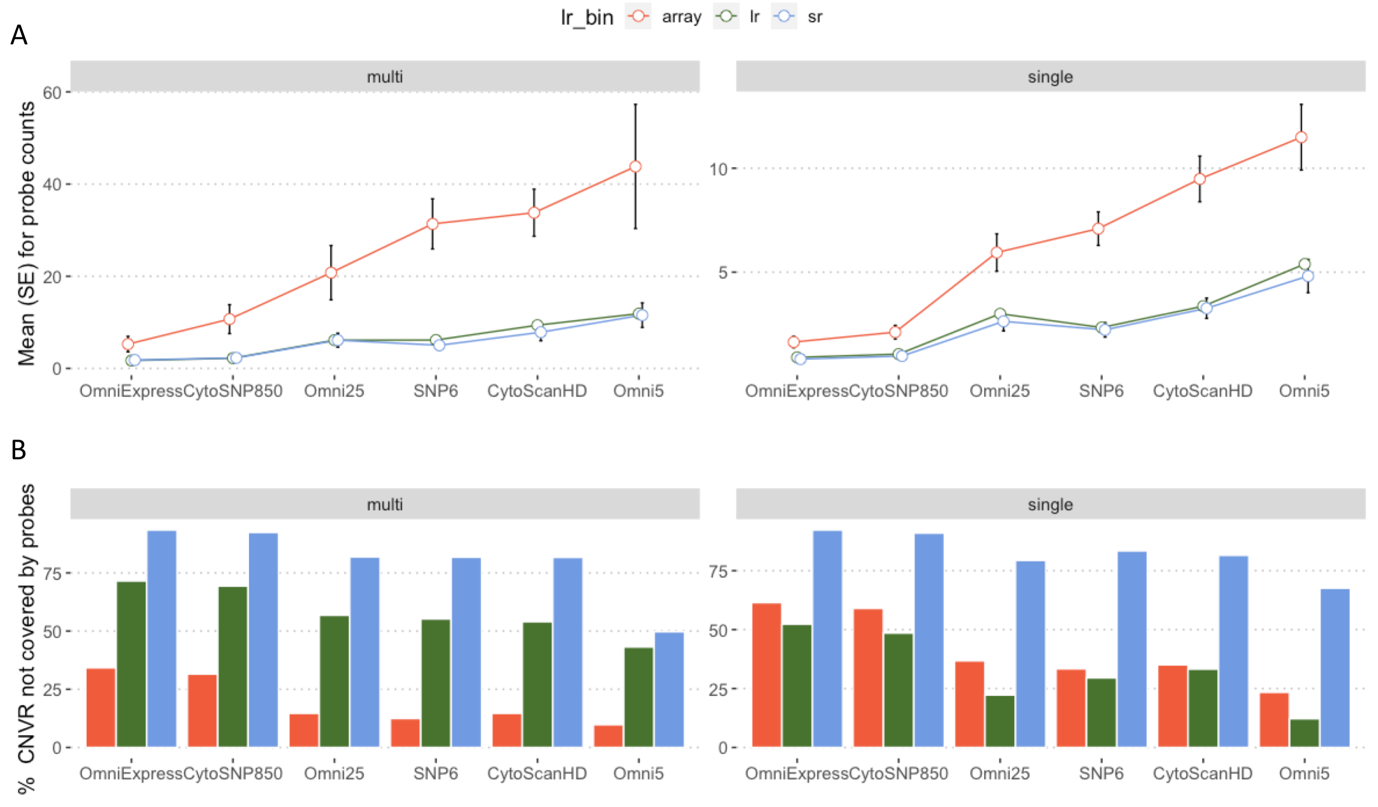


Figure S2. **Array probe coverage and within technology support.** A. Mean and SE interval around it for the number of probes spanned by CNVR from array (red), long reads (green) and short reads (blue). Left and right panels correspond to multi-dataset supported CNVRs and singletons (single dataset). On the x-axis selected chips for which the probe coverage is calculated are shown. The multi-supported CNVRs have consistently higher probe coverage in all chip designs; B. Percent of CNVRs which are not covered by any probes, following the same color code as on the top panel. Short-read derived CNVR dominate with the least coverage for both multi-supported and singleton ones. Long-read CNVRs show a trend, opposite of array-based ones in that the multi-supported long-read CNVRs tend to be devoid of probes more often than the respective singletons.

Figure S3. **Composition and percentages for CNV loci, per long read quality bin.**
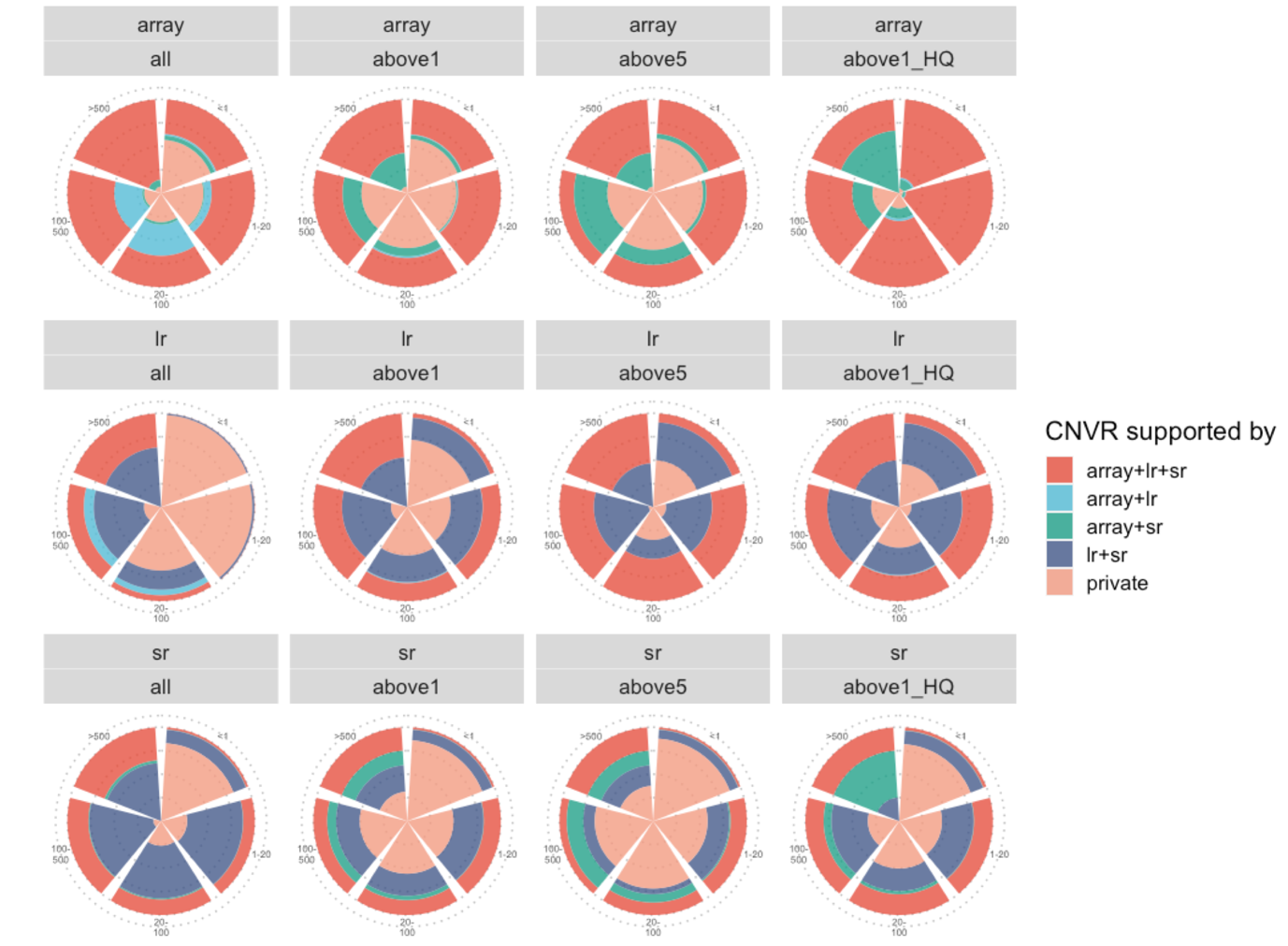
 Percentages of CNV loci split by the combinations of supporting technoogies (color legend to the right). The technologies are presented in rows, from top to bottom – array, long reads, short reads. The binning described in the results section two is represented in columns, in short, “all” – unfiltered original dataset; “above 1” – CNV loci resulted in the datase in which the long-read CNVRs with score >1; “above5” – the same as previous, but score >5; “above1_HQ” – in addition to long-read CNVR, both array and short-ead CNVRs are included if and only if they are classified as High Quality (HQ), as defined by the duphold tool score. The segmens in the “cake” show different sizes of CNV loci.


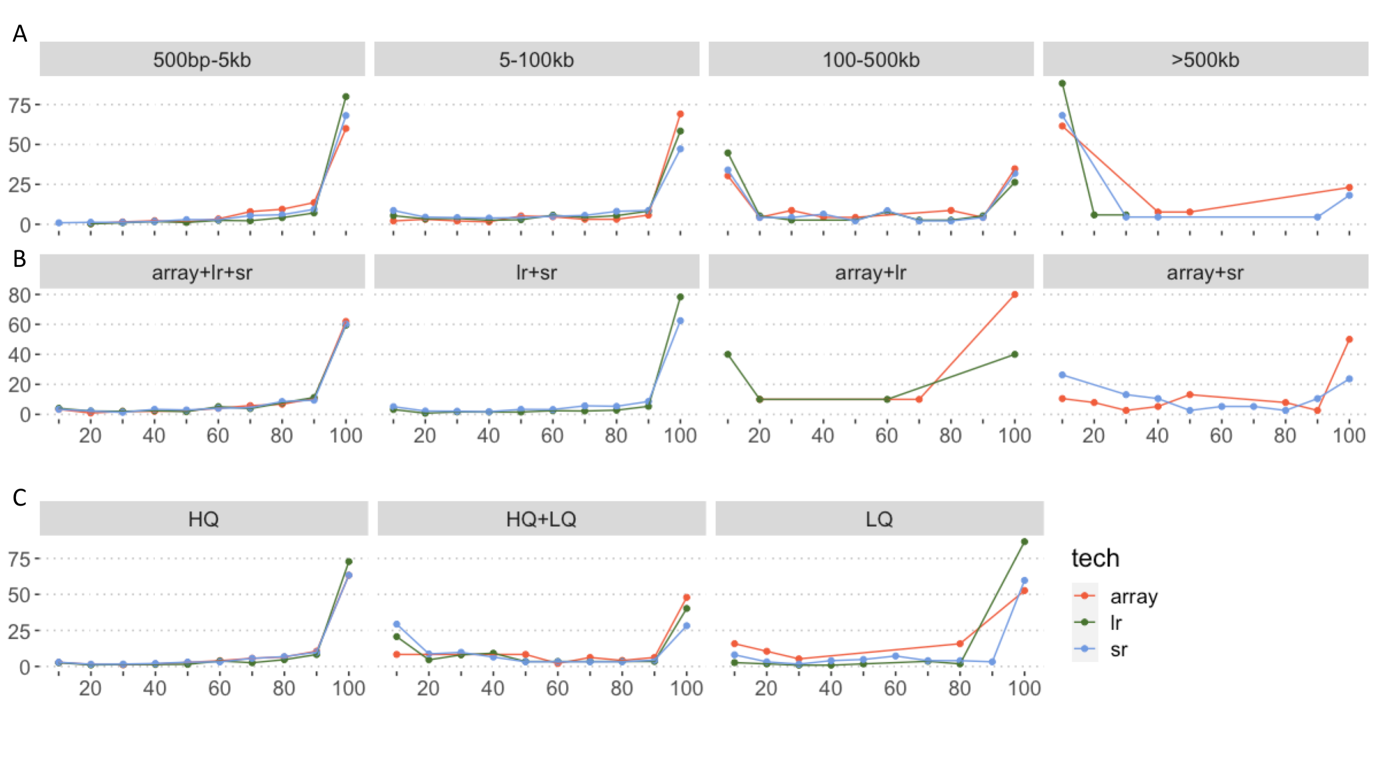
Figure S4. **Composition and percentages for CNV loci, per long read quality bin.** Line plots reflecting the percentages (x-axis) of CNV loci split by: A. CNV loci size bins; B. Combination of technologies supporting a CNV locus; C. Quality tags of CNVRs constituting the CNV loci (only HQ, only LQ or both). Color legend: array-derived CNV loci (red), long-read CNV loci (green) and short read CNV loci (blue)


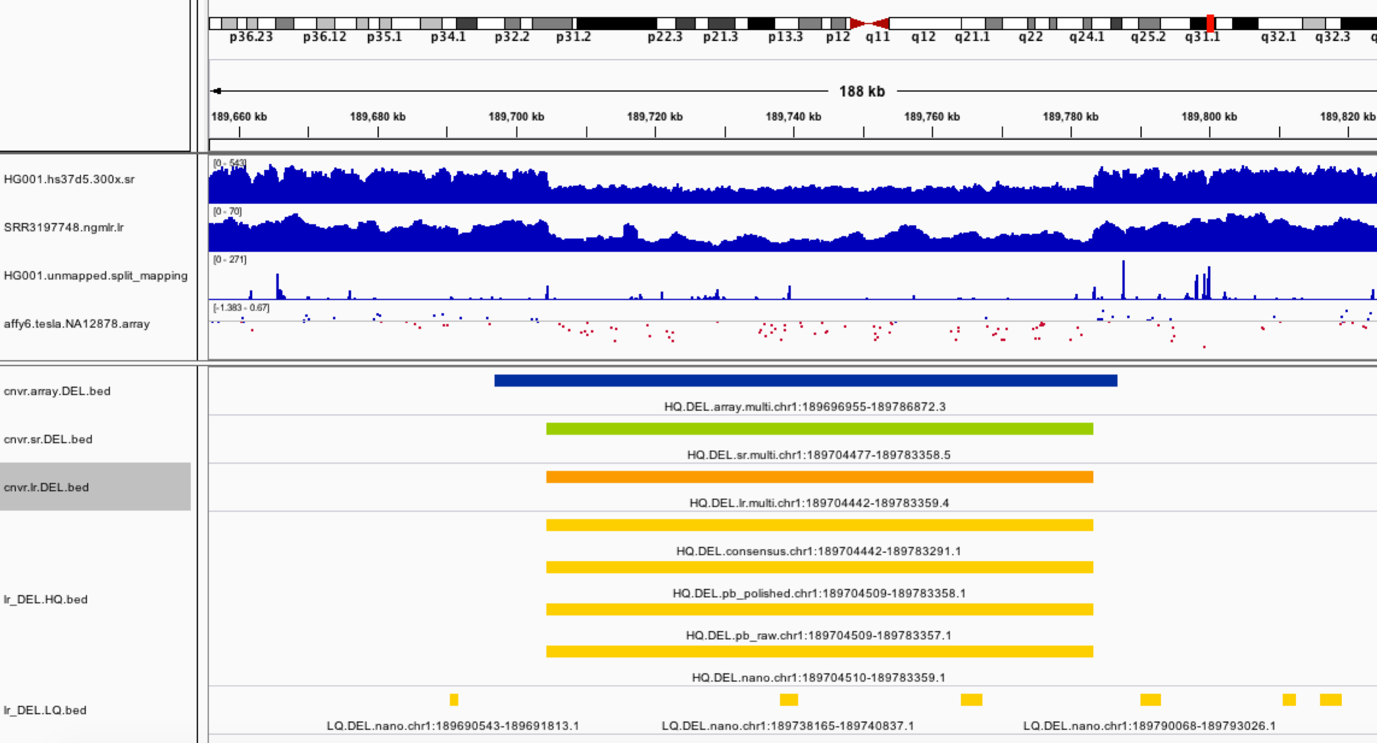


Figure S5. **Example deletion with multiple evidences from all technologies.** IGV desktop screenshot of a genomic region with predicted deletion. Tracks from top to bottom: Short-read sample coverage, long-read sample coverage, short-read discordant reads coverage, Affymetrix SNP6.0 array signal data; array CNVR (blue); short-read CNVR (green); long-read CNVR (orange); CNV calls from long-read datasets (light orange), split to High Quality (HQ) and Low Quality (LQ)


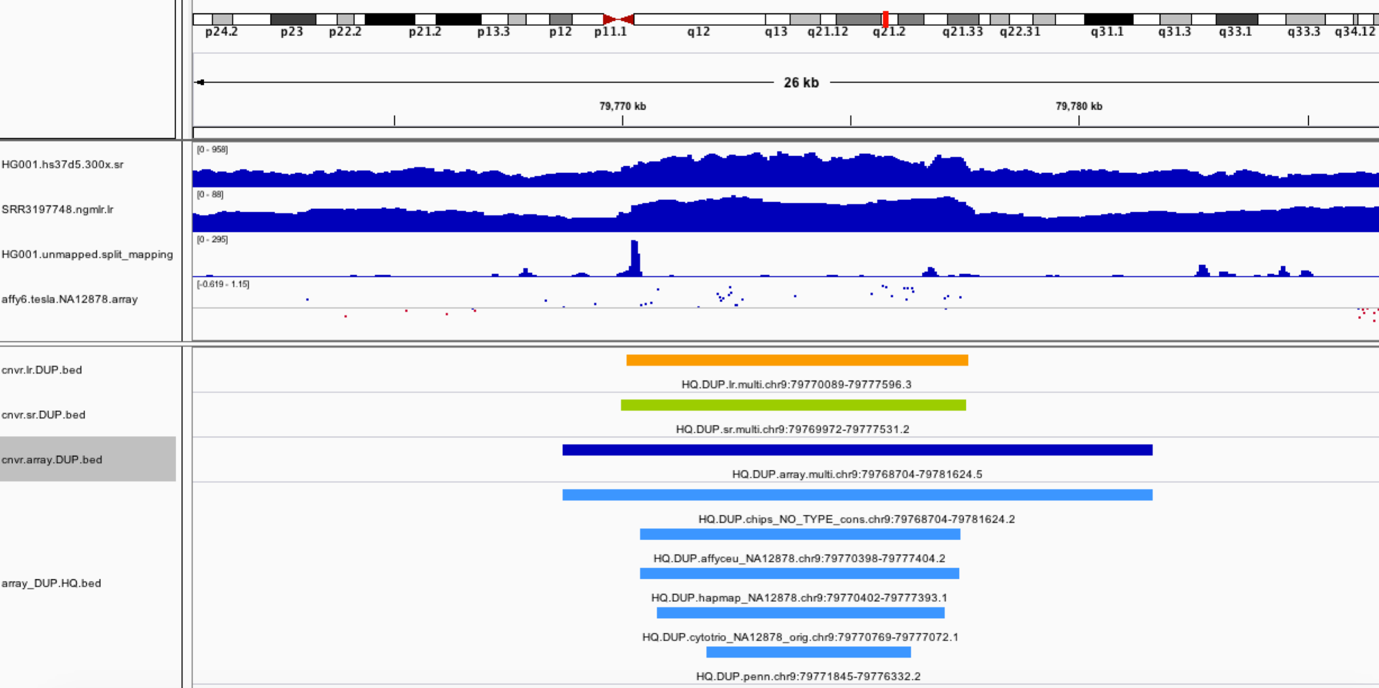


Figure S6. **Example duplication with multiple evidences from all technologies.** IGV desktop screenshot of a genomic region with predicted deletion. Tracks from top to bottom: Short-read sample coverage, long-read sample coverage, short-read discordant reads coverage, Affymetrix SNP6.0 array signal data; long-read CNVR (orange); short-read CNVR (green); array CNVR (blue); CNV calls from array datasets (light blue), split to High Quality (HQ) and Low Quality (LQ), only HQ segments are present in this locus.


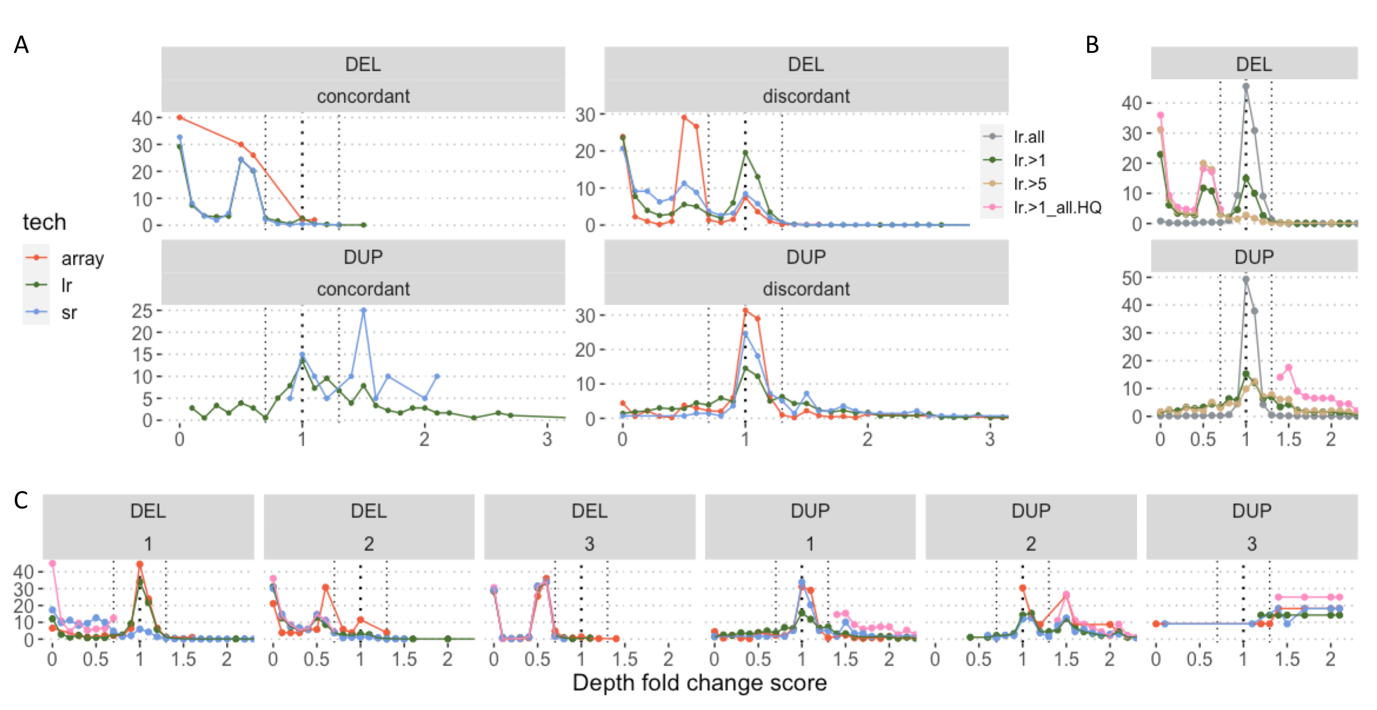


Figure S7. **Short read score distribution across various metrics.** Short-read duphold score (x-axis) and line plot of percentages (y-axis) for CNVRs split by: A. Long-read predicted support (VaPoR), where “concordant” means that the chosen long-read dataset supports presence of CNV while “discordant” label means the lack of such support ; color legend to the left; B. Long-read only CNVRs binned by long-read score; color legend to the left; C. Number of technologies supporting a CNVR (1, 2 or 3), long-read CNVRs are presented by both green (score >1) and pink (score > 5) categories, array CNVRs in red and short-read ones in blue.


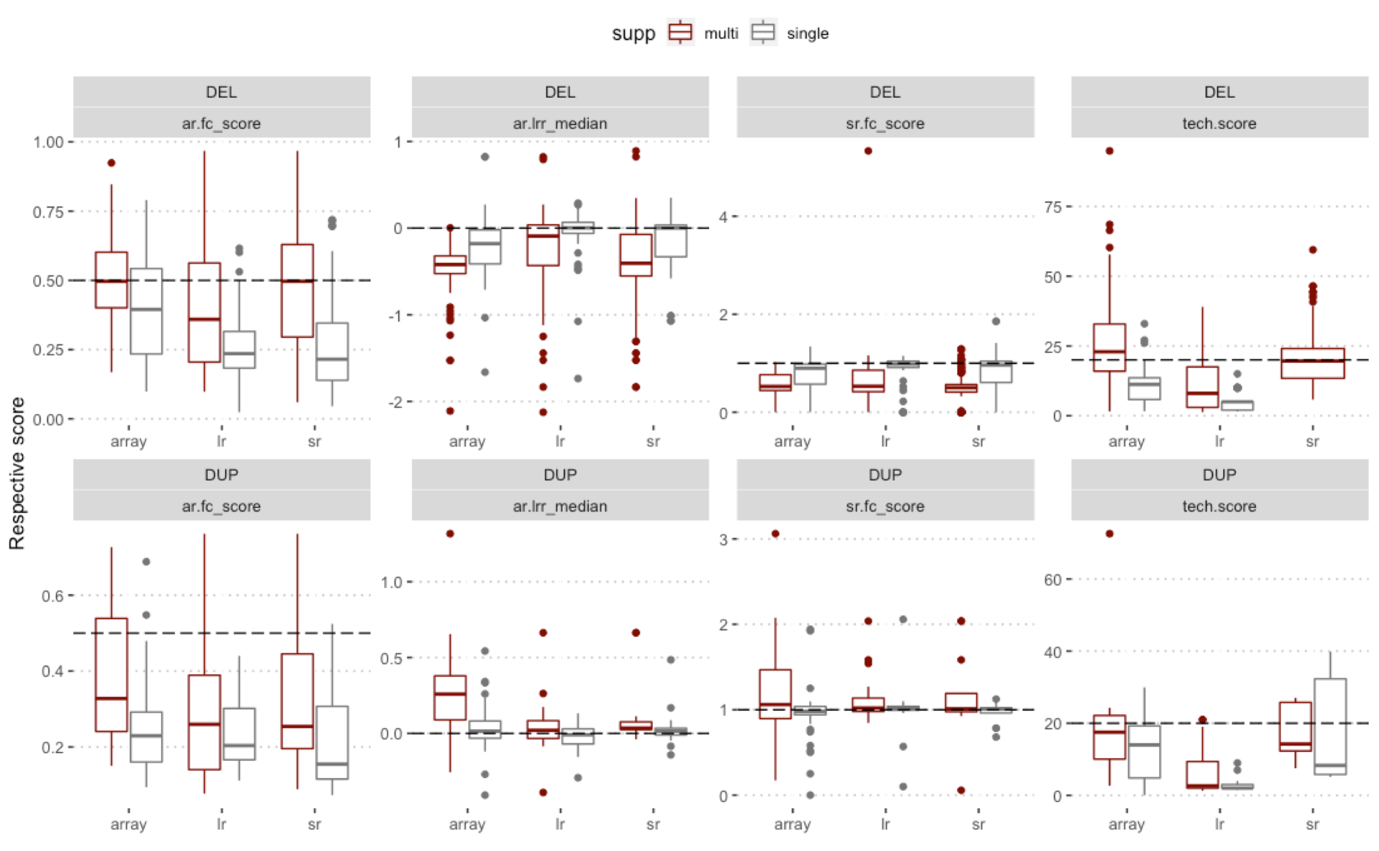


Figure S8. **Overview of various scores and relation to technology support.** The various collected scores distributions are shown (y-axis) across CNVRs in three technologies (x-axis), split to two categories of within-technology support (burgundy – supported by multiple datasets, gray – supported by a single dataset). Top and bottom rows for deletions and duplications respectively. From left to right: ar.fc_score – array fold change score; ar.lrr_median – median of array Log R Ratio; sr.fc_score – short-read fold change score, .i.e. duphold score; tech_score – score given by a chosen caller within each technology (array – PennCNV score, long reads – SVIM score, short reads – GRIDSS score).


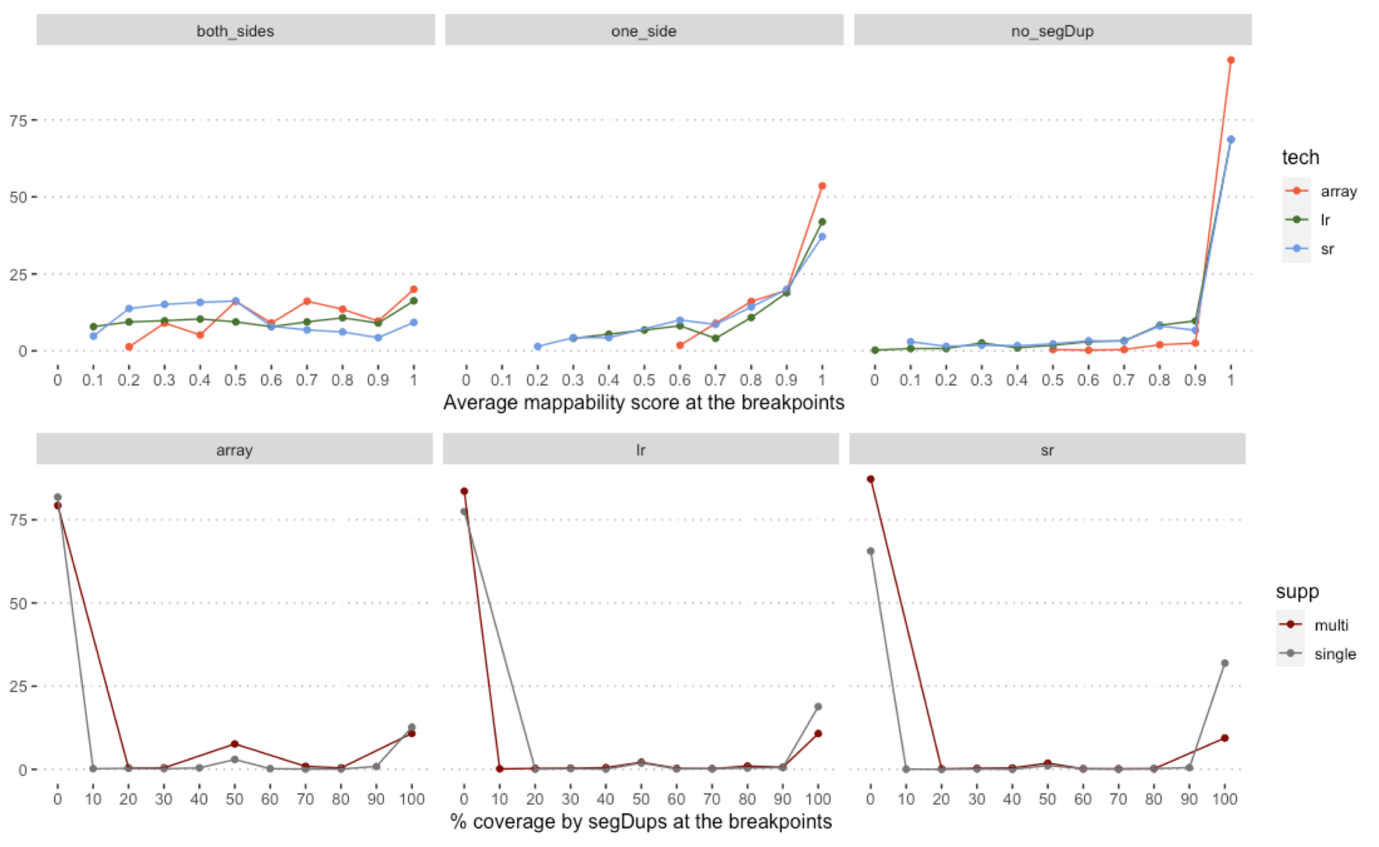


Figure S9. **Average mappability and coverage by segmental duplications at the CNVR breakpoints.** Sequence at +/- 100 basepairs around the predicted breakpoints is taken for the calculation. Top panel: x-axis – average mappability at the breakpoints, y-axis – line plot of percentages of CNVRs; left to right panels – overlap with segmental duplications (segDups) at both breakpoints, only one and no overlap; bottom panel: x-axis – coverage by segDups in percentage of the CNVR, y-axis line plot of percentages of CNVRs; left to right panel – technologies from array, to long reads to short reads. Color code: burgundy – supported by multiple datasets, gray – supported by a single dataset.


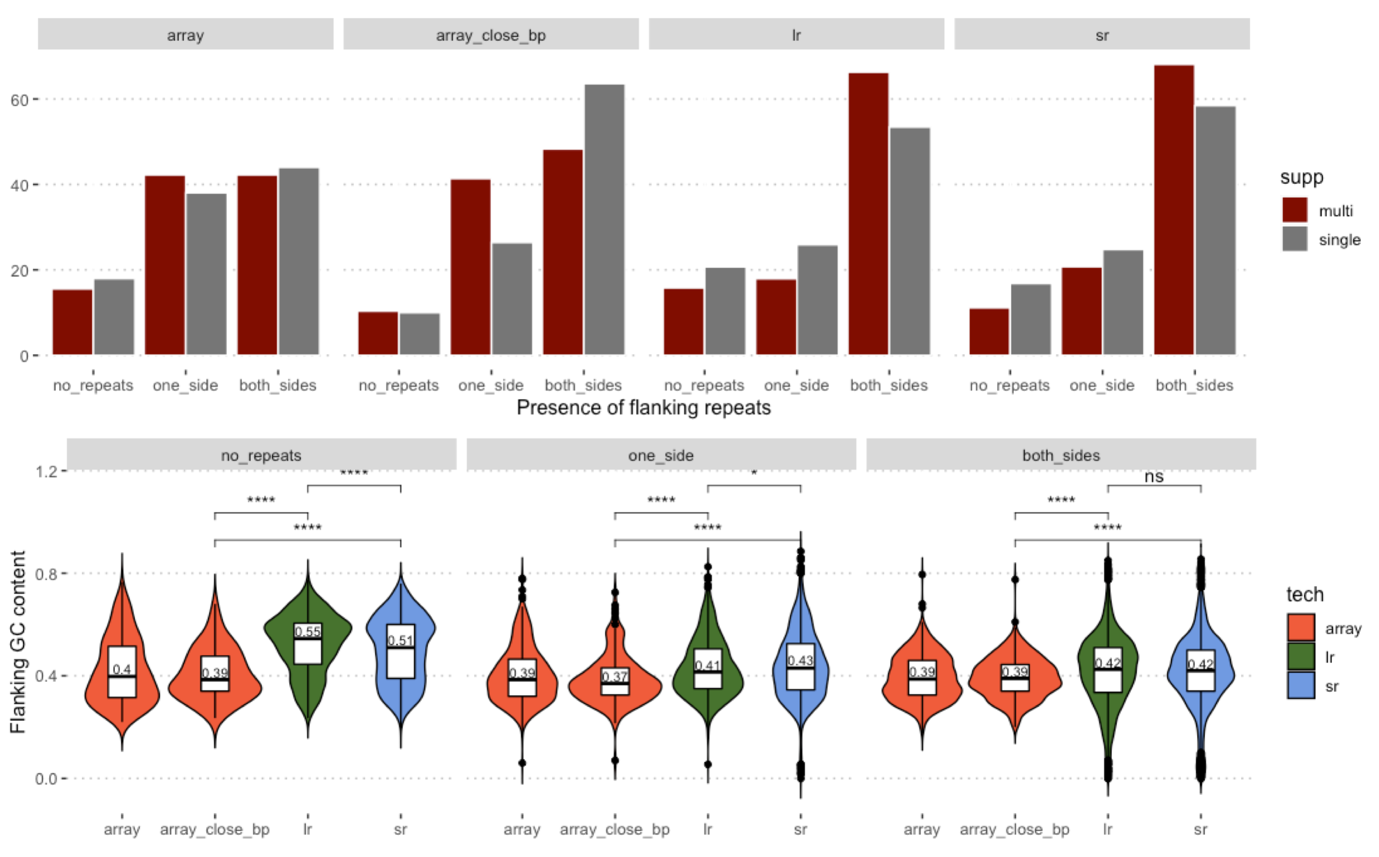


Figure S10. **Repeat and GC content of flanking regions of CNVs.** Sequence at +/- 100 basepairs around the predicted breakpoints is taken for the calculation. Top panel: Presence of repeats at the breakpoints on x-axis (no repeats, one side only, both sides); CNVRs split by within-technology support (burgundy – supported by multiple datasets, gray – supported by a single dataset). Percentage of CNVRs in each bin are shown (y-axis), panels left to right: all array CNVRs, subset of array CNVRs with breakpoints close to other technology breakpoints (assuming more accurate set of calls), long-read CNVRs, short-read CNVRs; bottom panel: the same sets as in the panels above on the x-axis, GC-content percentage at the breakpoints on the y-axis. Median value for each violin plot is shown, significant difference (Wilcoxon test) indicated by stars or ns for not significant.
